# Integrated Multiomic Analysis Reveals the High-Fat Diet Induced Activation of the MAPK Signaling and Inflammation Associated Metabolic Cascades via Histone Modification in Adipose Tissues

**DOI:** 10.3389/fgene.2021.650863

**Published:** 2021-06-28

**Authors:** Zhe Wang, Ming Zhu, Meng Wang, Yihui Gao, Cong Zhang, Shangyun Liu, Shen Qu, Zhongmin Liu, Chao Zhang

**Affiliations:** ^1^Department of Plastic and Reconstructive Surgery, Shanghai Institute of Precision Medicine, Shanghai Ninth People’s Hospital, Shanghai Jiao Tong University School of Medicine, Shanghai, China; ^2^Translational Medical Center for Stem Cell Therapy and Institute for Regenerative Medicine, Shanghai East Hospital, Shanghai Key Laboratory of Signaling and Disease Research, Frontier Science Center for Stem Cell Research, School of Life Sciences and Technology, Tongji University, Shanghai, China; ^3^Department of Endocrinology and Metabolism, National Metabolic Management Center, Shanghai Tenth People’s Hospital, School of Medicine, Tongji University, Shanghai, China; ^4^Department of Cardiac Surgery, Shanghai East Hospital, School of Medicine, Tongji University, Shanghai, China

**Keywords:** adipose tissue, epigenetics, high-fat diet, inflammation, methylation, obesity

## Abstract

**Background:**

The number of diet induced obese population is increasing every year, and the incidence of type 2 diabetes is also on the rise. Histone methylation and acetylation have been shown to be associated with lipogenesis and obesity by manipulating gene expression via the formation of repression or activation domains on chromosomes.

**Objective:**

In this study, we aimed to explore gene activation or repression and related biological processes by histone modification across the whole genome on a high-fat diet (HFD) condition. We also aimed to elucidate the correlation of these genes that modulated by histone modification with energy metabolism and inflammation under both short-term and long-term HFD conditions.

**Method:**

We performed ChIP-seq analysis of H3K9me2 and H3K9me3 in brown and white adipose tissues (WATs; subcutaneous adipose tissue) from mice fed with a standard chow diet (SCD) or HFD and a composite analysis of the histone modification of H3K9me2, H3K9me3, H3K4me1 and H3K27ac throughout the whole genome. We also employed and integrated two bulk RNA-seq and a single-nuclei RNA sequencing dataset and performed western blotting (WB) to confirm the gene expression levels in adipose tissue of the SCD and HFD groups.

**Results:**

The ChIP-seq and transcriptome analysis of mouse adipose tissues demonstrated that a series of genes were activated by the histone modification of H3K9me2, H3K9me3, H3K4me1, and H3K27ac in response to HFD condition. These genes were enriched in Kyoto Encyclopedia of Genes and Genomes (KEGG) pathways involved in lipogenesis, energy metabolism and inflammation. Several genes in the activated mitogen-activated protein kinase (MAPK) pathway might be related to both inflammation and energy metabolism in mice, rats and humans fed with HFD for a short or long term, as showed by bulk RNA-seq and single nuclei RNA-seq datasets. Western blot analyses further confirmed the increased expression of *MET*, *VEGFA* and the enhanced phosphorylation ratio of p44/42 MAPK upon HFD treatment.

**Conclusion:**

This study expanded our understanding of the influence of eating behavior on obesity and could assist the identification of putative therapeutic targets for the prevention and treatment of metabolic disorders in the future.

## Introduction

As one of the world’s greatest health challenges, obesity is usually accompanied by an increased risk for many chronic diseases, including type 2 diabetes, cardiovascular, hypertension and dyslipidemia. Obesity is a multifactorial disorder that is associated with both genetic and environmental factors, including eating behavior and exercise (Inagaki et al., 2016). As the link between genetic variants and environmental factors, the role of epigenetic modifications in obesity, including histone modifications, DNA methylation and non-coding RNA, had gained much attention in the late decade. Metabolic diseases, such as obesity and diabetes, were found to be tightly associated with epigenetic variation (Ling and Ronn, 2019). Among epigenetic locus, same histone could involve various modifications of several chemical groups, such as methylation and acetylation (Okuno et al., 2013), to activate or repress gene expression. For example, the various degrees of methylation of lysine 9 of histone H3 (H3K9me) are associated with transcriptional repression, whereas H3K4me and H3K27ac are associated with transcriptional activation.

Adipose tissues play vital roles in maintaining energy balance and are classified into white adipose tissue (WAT) and brown adipose tissue (BAT). WAT is responsible for energy storage and obesity syndrome, while BAT consumes fat to generate heat, which is essential for body weight and temperature control (Lee et al., 2014). The differentiation and stimulation of adipose tissue were regulated by various transcription factors and epigenetic modifications (Klose et al., 2006; Tateishi et al., 2009; Ohno et al., 2013; Zhou et al., 2014; Inagaki et al., 2016; Sanchez-Gurmaches et al., 2016; Wang and Seale, 2016; Cheng et al., 2018). For example, previous studies demonstrated the important roles of the methylation modifications of histone H3K9 sites in regulating metabolism of adipose tissue and maintaining homeostasis (Klose et al., 2006; Tateishi et al., 2009; Ohno et al., 2013; Cheng et al., 2018). High levels of H3K9me2 inhibited the expression of *PPAR*γ and thereby affected the process of adipogenesis from preadipocytes (Rosen and Spiegelman, 2001; Takada et al., 2009). The deletion of *G9a* with the methylation of H3K9me1 and H3K9me2 in mouse adipose tissues could increase the expression of *PPAR*γ and thereby promoted adipogenesis by increasing adipogenic gene expression (Tachibana et al., 2005; Wang et al., 2013). In addition, the histone demethylase *LSD1* (also known as *KDM1*) was essential for the initiation of adipogenesis by decreasing H3K9me2 levels and maintaining H3K4me2 levels at promoter regions (Musri et al., 2010; Duteil et al., 2014). Moreover, high-fat diet (HFD) could stimulate the proliferation of preadipocytes in subcutaneous adipose tissue (Joe et al., 2009). A long term HFD could induce changes in epigenetic modifications in WAT, as demonstrated by decreased levels of H3K4me2 and increased levels of H3K36me2 in mice with HFD induced obesity (Nie et al., 2017). These results suggested that changes in epigenetic states could disrupt the functions of adipose tissue and ultimately led to obesity.

Energy metabolic equilibrium and inflammation of adipose tissues are essential during the establishment of obesity syndrome. Usually, less than 10% of macrophages are located in normal adipose tissues, whereas the development of obesity is associated with increases in the number of macrophages and the polarization of M1 macrophages, which are closely related to energy homeostasis and inflammation (Odegaard and Chawla, 2008; Chawla et al., 2011). Macrophages are the most abundant immune cells and proinflammatory cytokines in obese adipose tissues, that could promote insulin resistance of adipose tissue and result in type 2 diabetes and other metabolic diseases (Odegaard and Chawla, 2008; Chawla et al., 2011). However, little is known on the correlation between genes controlled by histone modification and energy metabolism or inflammation of WAT and BAT in HFD induced obesity syndromes.

In this study, to understand the effect of histone modification on gene expression across the whole genome under high fat diet conditions, we analyzed the methylation modifications of histone H3K9 sites (H3K9me2 and H3K9me3) near the transcription start site and H3K4me1 and H3K27ac histone modifications throughout the genome. Because of the fact that the traditional bulk RNA sequencing cannot identify cell type specific heterogeneity due to the mixture of cell types in the specimen, we additionally integrated two bulk RNA-seq and a single-nuclei adipocyte RNA-seq datasets (SNAP-seq, GSE133486) (Rajbhandari et al., 2019) to identify the correlation between genes activated via histone modification and inflammation associated energy metabolism. In this study, we attempted to address the following questions: (1) Which genes are activated or repressed by histone modification in mouse adipose tissues under a long term HFD? (2) What biological processes are associated with genes that are modulated by HFD induced histone modifications? (3) Are there any correlations between genes modulated by HFD induced histone modifications and energy metabolism or inflammation under both short- and long-term HFD conditions? Elucidation of these questions will expand our understanding of the influence of eating behavior on obesity and could assist the identification of putative therapeutic targets for the prevention and treatment of metabolic disorders.

## Materials and Methods

### Animal Treatment

All animal experiments were approved by the Institutional Animal Care and Use Committee (IACUC) of the Shanghai Jiao Tong University and the Tongji University. Six-week-old wild-type C57BL/6J mice were fed either a standard chow diet (SCD) or a HFD (D12497, 60 kcal% fat, Research Diets). The body weight was monitored once a week. After 12 weeks, the BAT and WAT of the mice were obtained for subsequent H3K9me2 and H3K9me3 chromatin immunoprecipitation (ChIP-seq) assay.

### ChIP-seq of H3K9 Methylation

The BAT and WAT of mice were removed and cleaved into small pieces. Formaldehyde was added to a final concentration of 1% and rotated at room temperature for 8 min. Glycine was added to a final concentration of 125 mM to terminate the cross-linking reaction, and the sample was rotated at room temperature for additional 10 min. H3K9me2 (Anti-Histone H3 (tri methyl K9) antibody, ChIP Grade, ab8898) and H3K9me3 (Anti-Histone H3 (di methyl K9) antibody [mAbcam 1220], ChIP Grade, ab1220) were used for immunoprecipitation assay. Libraries were prepared using the KAPA Hyper Prep Kit following the manufacturer’s instructions and sequenced with the Illumina HiSeq X Ten system.

The sequence quality was assessed using FastQC v0.11.9, and adaptors were trimmed using trim_galore v0.6.6. After the data were filtered, the DNA sequences were aligned to the reference genome (mm9) using Bowtie2 (v2.4.2)(Langmead and Salzberg, 2012), and the reads that mapped to multiple sites were removed. Next we performed model-based analysis of ChIP-seq using MACS2 (Zhang et al., 2008) for enriched-region identification (peak calling), and determined the location in the genome with differentially enriched peaks (*P* < 0.05) between the SCD and HFD fed mice. The enriched peaks were annotated with the R package ChIPseeker (Yu et al., 2015). Genes that showed different-sized peaks of H3K9me2 or H3K9me3 in the 3 kb upstream and downstream of the transcription starting sites (defined as the promoter regions) between the SCD and HFD fed mouse adipose tissue were selected for subsequent KEGG enrichment analysis, which was conducted with the R package clusterProfiler (Yu et al., 2012).

### Integrated Analysis of H3K4 and H3K27 ChIP-seq Datasets

To clarify the effect of histone modification on gene expression under HFD conditions, we further integrated H3K4me1 and H3K27ac ChIP-seq data (GSE132885) of mouse WAT that was fed with SCD or HFD for 8 weeks (Caputo et al., 2020). From processed data in a published paper (Caputo et al., 2020), we obtained H3K4me1 and H3K27ac enriched regions in subcutaneous adipose tissue of SCD or HFD fed mice using bigWigtoBedGraph v377. The R package edgeR (McCarthy et al., 2012) was used to determine the differences of the number of enriched peaks in gene promoters across the whole genome between SCD and HFD fed mouse adipose tissues. The KEGG enrichment analyses were conducted as mentioned above.

### Integrated Analysis of Bulk RNA and Single-Nuclei RNA Sequencing Data of Adipose Tissue

To illustrate the correlation between genes activated by HFD via histone modification and inflammation, we integrated two bulk RNA sequencing datasets (GSE69607 and GSE158627) in this study (Jablonski et al., 2015; Zhang X. et al., 2020). Using data from GSE69607, we compared the differentially expressed genes between M1 and M2 macrophages using the R package limma (Ritchie et al., 2015), and genes with a *P*-value < 0.05 and logFC > 0.58 were identified as macrophage markers. The genes that served as macrophage markers and also showed differential peaks (in the promoter regions) between SCD and HFD fed mouse WAT (based on H3K9me2 ChIP-seq) were subjected to KEGG enrichment analysis. In addition, using data from GSE158627, we compared the differentially expressed genes between macrophages from WT (Lyz2-Cre) mice fed a SCD and HFD, which might be related to HFD induced inflammation in adipose tissue.

To further illustrate the correlation between genes activated by HFD via histone modification and energy metabolism at single cell resolution, we additionally integrated a single-nuclei adipocyte RNA sequencing dataset (SNAP-seq, GSE133486) in this study (Rajbhandari et al., 2019). We obtained the top 2000 variable genes from the metabolically active adipocyte subtype using the R package Seurat v3 and conducted a KEGG enrichment analysis with the R package clusterProfiler. In addition, we colored several genes (*MAP3K5*, *MAP3K14*, *MET*, and *VEGFA*) on the UMAP dimensional reduction plot to visualize the gene expression level and the number of expressed genes detected in single adipose cell using the R package Seurat v3.

To confirm the activated mitogen-activated protein kinase (MAPK) signaling pathway in adipose tissue of obese rat and human, we analyzed two bulk RNA sequencing datasets from GSE142401 and GSE1813. The differentially expressed genes were calculated as above and the R package pheatmap v1.0.12 and clusterProfiler v3.12 were used for visualization.

### Analyses of Western Blot

To confirm the activated MAPK signaling pathway and the increased gene expression in HFD adipose tissues, we further conducted experimental validation using western blot. The adipose tissues of the rats were lysed in RIPA buffer (P0013B, Beyotime, China) supplemented with protease and phosphatase inhibitor cocktails (C0001 and C0004, Targetmol, United States) on ice. Total lysate was subjected to SDS-PAGE and electro-transferred to a PVDF membrane (IPFL85R, Merck, Germany). The membranes were blocked with 5% BSA, incubated with primary antibody (Supplementary Table 1) and HRP-conjugated secondary antibody. Tanon chemiluminescence image detection system (5200S, Tanon, China) was used to detect the luminescent signals. Statistically differences were evaluated by unpaired student’s *t* test. GraphPad Prism software (La Jolla, CA, United States) was used for data analysis and visualization.

## Results

### Various Signaling Pathways Are Activated by Histone Modifications in Mice Adipose Tissues Upon HFD Treatment

**FIGURE 1:**
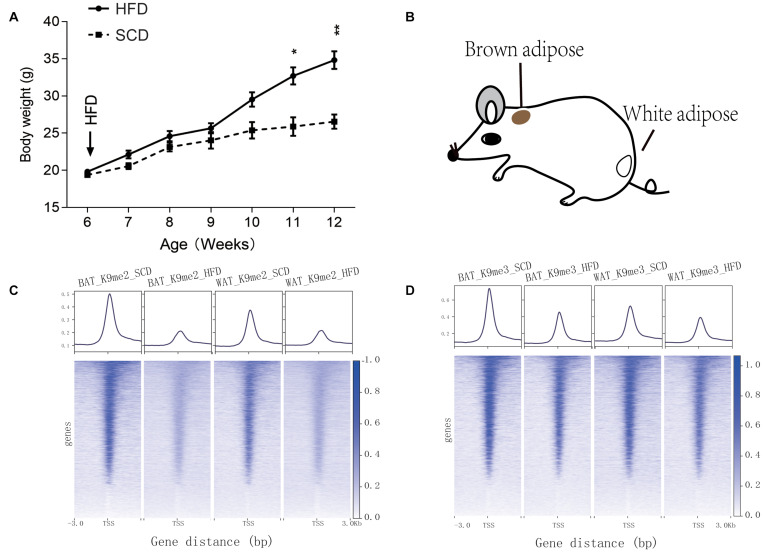
Information of SCD and HFD mice used in this study and peak enrichment of H3K9 methylation. **(A)** Body weight monitoring of mice under control (SCD) and high fat diet (HFD) treatments. *P < 0.05, **P < 0.01. **(B)** Dissection location of mouse brown adipose tissue and white adipose tissue used for the H3K9me2 and H3K9me3 ChIP-seq experiments. **(C,D)** Peak enrichment at histone H3K9 methylation modification (H3K9me2 and H3K9me3) near the transcription start site (TSS, ±3 kb) of genes.

**FIGURE 2:**
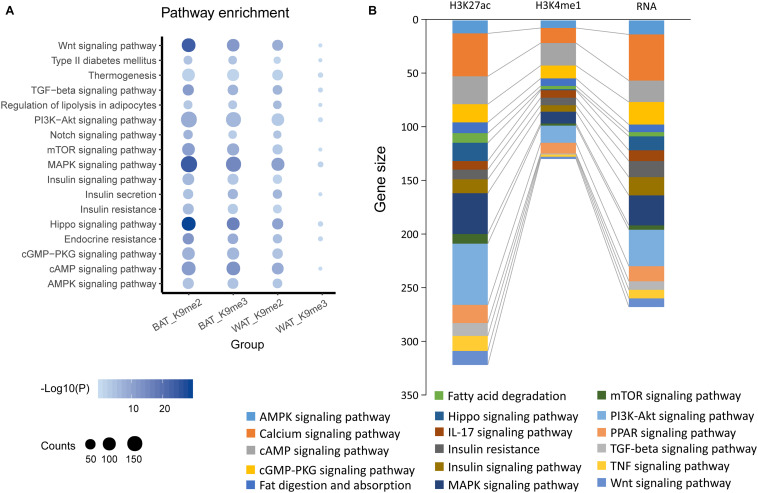
KEGG pathway enrichments of BAT or WAT under SCD and HFD conditions. **(A)** KEGG pathway enrichment by genes showing differential peaks in promoter regions identified by H3K9me2 (K9me2) or H3K9me3 (K9me2) ChIP-seq of mouse brown adipose (BAT) or white adipose tissues (WAT) under HFD condition compared with SCD conditions. **(B)** KEGG pathway enrichment of H3K27ac, H3K4me1 and RNA-activated genes in brown adipose (BAT) or white adipose tissue (scWAT) under HFD condition compared with SCD conditions.

Starting at 6-week age, wild type mice were fed with either SCD or HFD, and a significant difference (*P* < 0.05) in body weight began to be observed at week 11 (Figure 1A). On week 12, the BAT and WAT of the mice (Figure 1B) were dissected to examine the differential methylation modifications of H3K9me2 and H3K9me3. Our results showed that high fold enrichment peaks appeared at the promoter regions (Figures 1C,D). Noticeably, the peak fold enrichment of H3K9me2 and H3K9me3 modifications was decreased in both the BAT and WAT of HFD fed mice compared with those of SCD fed mice (Figures 1C,D). These results suggested a decreased level of H3K9 methylation crossing the whole genome in HFD fed mice compared with SCD fed group. The KEGG enrichment analysis revealed that the genes with H3K9 demethylation modification in adipose tissue of the HFD-fed mice were enriched in pathways related to lipogenesis, energy metabolism, immunity and inflammation (Figure 2A). Among these pathways, the MAPK signaling pathway was enriched with a highest number of genes and had a *P*-value less than 0.001 (Figure 2).

We also analyzed published data on histone modifications, including H3K27ac and H3K4me1 ChIP-seq and bulk RNA-seq data (GSE132885). These data showed that compared with the SCD fed mice, many genes in mouse adipose tissue were activated by the consumption of a HFD for 8 weeks. These genes were also enriched in pathways similar to those found for H3K9 (Figure 2B), including the MAPK signaling pathway. All of these results suggested that the changes in histone modifications induced by a HFD could activate various genes that are related to the biological processes of lipogenesis, energy metabolism, immunity and inflammation and include the MAPK signaling pathway.

### Activation of Genes in the MAPK Signaling Pathway via Histone Modification Are Related to Inflammation and Energy Metabolism

**FIGURE 3:**
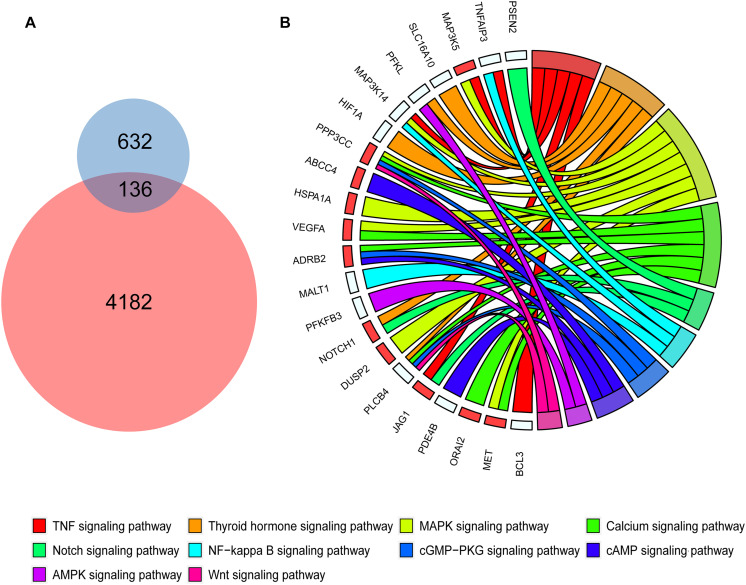
Polarized macrophage marker genes activated by HFD and relevant KEGG pathways. **(A)** The Venn diagrams shows 136 genes shared between the polarized macrophage marker genes and activated genes with differential peaks in the promoter determined by H3K9me2 ChIP-seq data in mouse white adipose tissue of HFD conditions. **(B)** The chord diagrams show the KEGG pathways enriched by the 136 shared genes. The red squares represent the genes shared between the 136 genes and differentially expressed genes of macrophages under SCD and HFD conditions.

To investigate whether genes with differential peaks caused by histone modification under SCD and HFD conditions exhibited correlations with inflammation and energy metabolism, we integrated two bulk RNA-seq datasets and a SNAP-seq dataset in this study. These RNA sequencing data involved macrophage polarization, which was important for macrophage inflammation and energy metabolism in adipose tissues. First, we found 768 genetic markers of M1 macrophages from the published bulk RNA dataset GSE69607 that showed higher expression levels in M1 macrophages than other types. Among these marker genes, 136 genes were also activated via histone modification in WAT under HFD feeding, as demonstrated by H3K9me2 ChIP-seq (Figure 3A). KEGG enrichment analyses showed that the 136 genes were enriched in some pathways, including the MAPK signaling pathway (Figure 3B). Among these genes, *Map3k5*, *Ppp3cc*, *Hspa1a*, *Vegfa*, *Dusp2*, and *Met* were also upregulated in macrophages of adipose tissue from HFD fed mice compared with the control group (red squares in Figure 3B, GSE158627), suggesting a strong correlation of MAPK signaling to inflammation upon HFD treatment.

**FIGURE 4:**
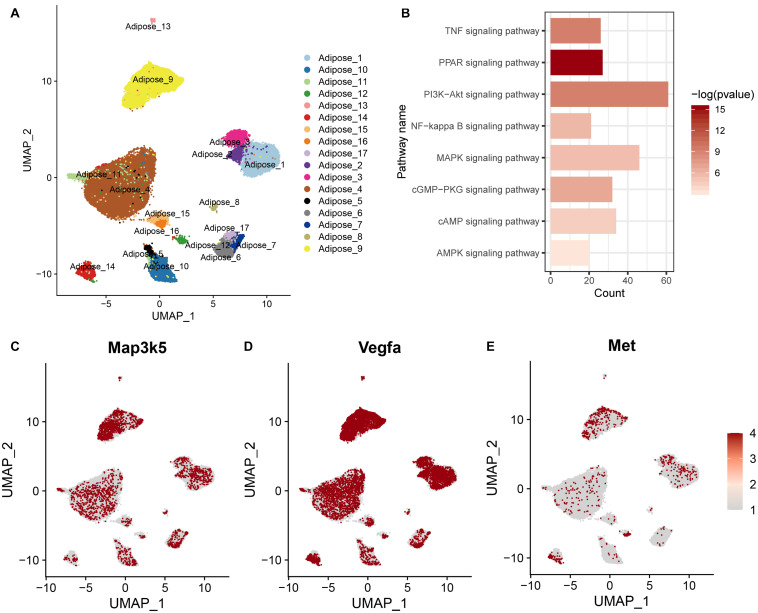
Gene expression and the related KEGG pathway in metabolic active adipocyte subtype by single-nuclei RNA sequencing. **(A)** UMAP plot of single-nuclei RNA sequencing data (GSE133486) illustrating the cell types of adipocytes. **(B)** KEGG pathway enriched by the top 2000 variable genes from the metabolic active adipocyte subtype. **(C–E)** Scaled expression levels of the *Map3k5*, *Met*, and *Vegfa* genes in the adipocyte subtype depicted using a red gradient (gray denotes a lack of expression).

In addition, we included a published single-nuclei RNA sequencing data of adipocytes (GSE133486) and defined a metabolic active adipocyte subtype in WAT (Adipose_9 in Figure 4A). We obtained the top 2000 variable genes from the metabolically active adipocyte subtype and assessed their enrichment in KEGG pathways, including the AMPK, cAMP, GMP-PKG, and MAPK signaling pathways (Figure 4B). We also found that the *Map3k5*, *Met*, and *Vegfa* genes were highly expressed in the metabolic active adipocyte subtype (Adipose_9), as reported in the published study (Figures 4C–E). Altogether, these results suggested that the *Map3k5*, *Met*, and *Vegfa* genes in the MAPK signaling pathway activated by HFD induced epigenetic modification might be related to both inflammation and energy metabolism, which pointed out an implication for better understanding of the effect of diet and eating behavior on obesity and energy homeostasis.

### Activated MAPK Signaling Pathway in Humans and Rats Under Short- and Long-Term HFD Conditions

**FIGURE 5:**
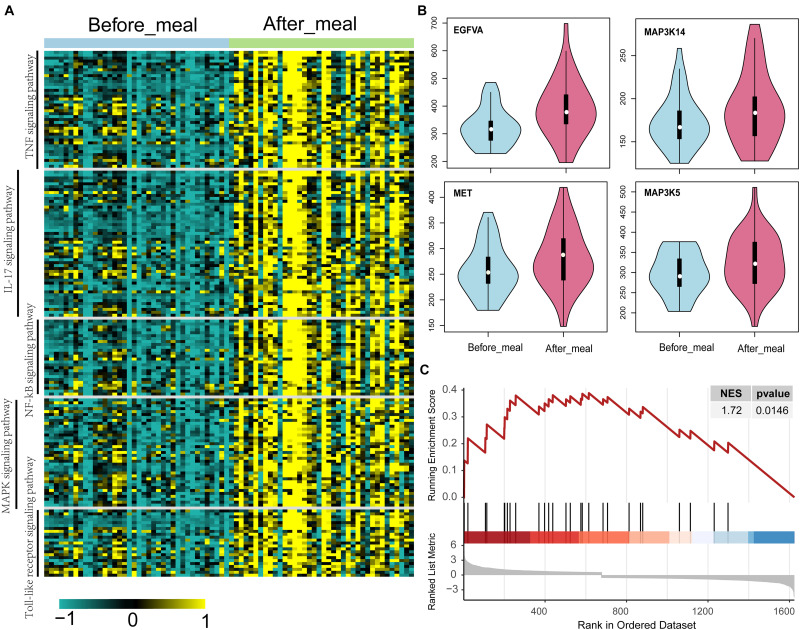
Up-regulated genes and activated KEGG pathways in humans and rats under short- and long-term HFD conditions. **(A)** KEGG pathways of the significantly differentially expressed genes between adipose tissue under fasting (0 h) and at 4 h post-meal condition. **(B)** Violin plots show the level of gene expression in adipose tissue under fasting (0 h) and at 4 h post-meal condition. **(C)** GSEA plot shows the genes upregulated in the adipose tissue of HFD rat are enriched in MAPK signaling pathway.

**FIGURE 6:**
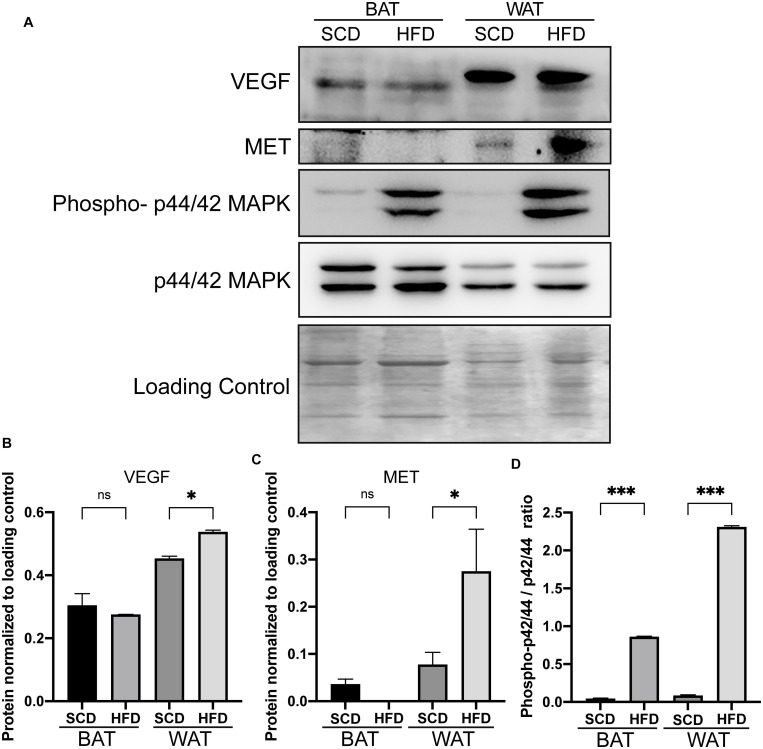
Increased expressions of genes in the adipose tissue of rats under HFD conditions by western blot analysis. Western blot analysis of the increased expressions of *Met* and *Vegfa* gene and the enhanced ratio of phosphorylation of p44/42 MAPK in adipose tissues from HFD rat. **(A)** The protein expression of Met, Vegfa and phospho-p44/42 MAPK in the adipose tissue of rats. **(B–D)** Bar plot of the statistical significance of *Met* and *Vegfa* expression and phosphorylation-p44/42 MAPK ratio in HFD and SCD adipose tissues in rat by unpaired student’s *t* tests. Bars represent standard errors. ^∗^, ^∗∗^, and ^∗∗∗^ indicates *P-*value < 0.05, 0.01, and 0.001, respectively. WAT, white adipose tissue; BAT, brown adipose tissue.

Based on the transcriptome of adipose tissue in humans under fasting conditions (0 h) and 4 h post-meal (GSE142401), we found that the genes that were upregulated after the meal were enriched in various KEGG pathways, including the IL-17, NF-kB, MAPK, Toll-like receptor and TNF signaling pathway (Figure 5A). In addition, several genes (*MAP3K5*, *MET*, and *VEGFA*) in the MAPK signaling pathway showed relatively higher expression levels after a high-fat meal than fasting condition (Figure 5B). Similarly, we found that these genes upregulated in HFD rat were also enriched in MAPK signaling pathway (Figure 5C).Furthermore, western blotting (WB) analyses showed increased expression of *Met*, *Vegfa* in WAT from HFD fed rat (Figures 6A–C). Additionally, the ratio of p44/42 MAPK phosphorylation was significantly upregulated in the HFD fed group (Figures 6A,D), which suggested a relatively activated state of MAPK pathway in obese adipose tissues. All of these results suggested that the MAPK signaling pathway could be activated by histone modification in adipose tissue after both long-term and short-term consumption of a HFD in house mice, rats and humans. The *MAP3K5*, *MET*, and *VEGFA* genes might be involved in inflammation associated energy metabolism via the activation of MAPK signaling cascades.

## Discussion

The post-transcriptional modifications of histone tails are essential for the regulation of the chromatin state, which could result in activation or repression of the transcription of the associated genes. For example, the di-methylation and tri-methylation of histone H3 at lysine 9 (H3K9me2 and H3K9me3) are mostly associated with gene repression, whereas histone H3K27 acetylation and H3K4me1 are features of gene activation (Zhang T. et al., 2020). During the growth and differentiation of adipose tissue, histone modifications not only determine the fate of precursor cells but also participate in the regulation of physiological activities and the body’s adaptation to the environment. In this study, H3K9me2 and H3K9me3 modification sites were analyzed in the adipose tissue of SCD and HFD fed mice by chromatin immunoprecipitation and high throughput sequencing. Generally, we found a stronger signal of peaks around the transcription start site in SCD fed mice than HFD-fed group (Figures 1C,D), suggesting the common demethylation and activation of transcription through the whole genome induced by HFD.

The KEGG analysis showed that the genes activated by HFD feeding were mainly associated with lipogenesis, energy metabolism and insulin resistance. For example, the Wnt (Bowers and Lane, 2008), mTOR (Caron et al., 2015) and Hippo signaling pathway (Shu et al., 2020) were major regulators of adipogenesis. The mTOR (Caron et al., 2015), cAMP, AMPK, and PI3K-Akt signaling pathway were closely related to energy metabolism.

Notably, we found that the MAPK signaling pathway was enriched with many H3K9me2 and H3K9me3 de-methylation activated genes (Figure 2A) as well as H3K4me1, H3K27ac and RNA expression activated genes (Figure 2B) upon HFD treatment. MAPK pathways regulated multiple cellular functions, including cell proliferation, migration, differentiation, and apoptosis. A previous study showed that MAPK signaling related genes interacted with dietary factors involved in inflammation and oxidative stress (Slattery et al., 2013). Inflammation of fat adipose tissue could induce insulin resistance and metabolic disease (Acin-Perez et al., 2020), which suggested that activated MAPK might help to improve the understanding of the epigenetic effects of HFD on obesity and metabolic disease.

To illustrate the correlation between obesity, energy metabolism and inflammation, we integrated bulk RNA-seq and single-nuclei RNA-seq dataset of adipocytes from other published study. Single-nuclei RNA-seq could show cell type specific differential gene expression and the results showed that some genes (*Map3k5*, *Met*, and *Vegfa*) in the MAPK signaling pathway activated by a HFD via histone modifications showed high expression levels in the metabolic active adipocyte subtype in WAT though single-nuclei RNA sequencing. These genes might also participate in inflammation (Figures 4, 5) in mice, rat and humans under short-term or long-term HFD (Figure 5). In addition, western blot further confirmed the higher expression of *Met* and *Vegfa* genes and the enhanced ratio of p44/42 MAPK phosphorylation under HFD conditions (Figure 6).

Previous studies also showed the potential role of *Map3k5*, *Met*, and *Vegfa* in obesity and metabolic dysregulation (Rudich et al., 2007; Bian et al., 2010; Lu et al., 2012; Elias et al., 2013; Sundaram et al., 2014a, b; Hattori et al., 2016; Haim et al., 2017). For example, Sundaram et al. (2014a, b) found that the expression of *Met* increased in obese mice with breast cancer compared with their lean controls, suggesting that *Met* was potentially associated with obesity and immunity (Sundaram et al., 2014a, b). The *MAP3K5* gene was upregulated in adipose tissues of obese animal (Haim et al., 2017), and a *MAP3K5* mutation that decreased RNA expression was linked to obesity with whole body insulin resistance (Rudich et al., 2007; Bian et al., 2010). In mice, *Map3k5* knockout mice became obese by interfering with brown fat function (Hattori et al., 2016). Vascular endothelial growth factor A (*Vegfa*), a member of the VEGF family, was also highly expressed in adipose tissues (Lu et al., 2012). Lu et al. (2012) found that mice with repressed expression of the *Vegfa* gene presented a lean phenotype and resistance to HFD induced body weight gain (Lu et al., 2012). Elias et al. (2013) showed that *Vegfa* overexpression in adipose tissue could enhance PGC-1α and UCP-1 expression, which resulted in increases in thermogenesis and energy expenditure and decreases in obesity, suggesting the important role of *Vegfa* under HFD conditions (Elias et al., 2013). In addition, increased *Vegfa* expression might be associated with higher inflammation in adipose tissues through its anti-inflammatory role in adipose tissue, which resulted in increases in activated macrophages (Elias et al., 2013; Schlich et al., 2013). Wang et al. (2006) found that the VEGF increased with the stimulation of pro-inflammatory factor TNFα in isolated progenitor cells of the human adipose, suggesting the potential roles of VEGF in inflammation. Here our study showed the significantly increased level of the phosphorylation of p42/44 MAPK in both BAT and WAT rat under a HFD condition, suggesting a relatively activated state of MAPK pathway in obesity adipose tissues. Overall, the demethylation activated genes *Map3k5*, *Met* and *Vegfa* in the activated MAPK signaling pathway could participate in inflammation and energy metabolism to lead to obesity, and thus, these genes might be promising markers for the prevention and treatment of metabolic diseases.

In summary, ChIP-seq technology was utilized to assess the changes in the modifications of histone H3 sites in BAT and WAT of mice under SCD or HFD conditions. We found that the HFD decreased the level of H3K9me2 or H3K9me3 level in gene promoters crossing the whole genome. Through integrated analyses of the bulk RNA transcriptome and single-nuclei adipocyte RNA sequencing in mouse, rat and human adipose tissues, we found that a series of genes that were activated by histone modification were also upregulated under both long-term and short-term HFD conditions. These genes, including *MAP3K5*, *MET*, and *VEGFA*, were enriched in KEGG pathways involving lipogenesis, energy metabolism, and inflammation pathways. All these findings would help us to improve the understanding of the influence of diet on obesity and human metabolic diseases through epigenetic modifications and could assist the identification of putative therapeutic targets for the prevention and treatment of metabolic disorders in the future.

## Data Availability Statement

The names of the repository/repositories and accession number(s) can be found below: https://www.ncbi.nlm.nih.gov/genbank/, GSE164530.

## Ethics Statement

All animal experiments were approved by the Institutional Animal Care and Use Committee (IACUC) of the Shanghai Jiao Tong University and the Tongji University.

## Author Contributions

ZW, MZ, CZh, and ZL conceived and designed the study. ZW, MZ, MW, CZo, SL, YG, and SQ performed the experiments. ZW, MZ, and YG performed the data analysis. All authors participated in data interpretation, manuscript drafting, and approved the final manuscript.

## Conflict of Interest

The authors declare that the research was conducted in the absence of any commercial or financial relationships that could be construed as a potential conflict of interest.
