# Supplementary material for: Integrated Multiomic Analysis Reveals the High-Fat Diet Induced Activation of the MAPK Signaling and Inflammation Associated Metabolic Cascades via Histone Modification in Adipose Tissues
Source: Front Genet. 2021 Jun 28;12:650863. doi: 10.3389/fgene.2021.650863 (PMC8273343; doi:10.3389/fgene.2021.650863)
Supplement: Supplementary file 1 [file Table_1.DOCX]

**Supplementary Table 1: Antibodies that we used for western blot analysis.**

| Antibodies | Cat No. | Brand | Dilution ratio |
| --- | --- | --- | --- |
| c-Met | 25869-1-AP | Proteintech | 1:1000 |
| VEGF | 19003-1-AP | Proteintech | 1:1000 |
| p44/42 MAPK (Erk1/2) | #4695 | Cell Signaling Technology | 1:1000 |
| Phospho-p44/42 MAPK (Erk1/2) | #9101 | Cell Signaling Technology | 1:1000 |
| HRP-Conjugated GAPDH Monoclonal Antibody | HRP-60004 | Proteintech | 1:10000 |
| HRP-Conjugated Beta Actin Monoclonal Antibody | HRP-60008 | Proteintech | 1:10000 |
